# Hip Replacement Surgery in 14-Year-Old Girl with Factor V Deficiency: Haemostatic Treatment and Thromboprophylaxis

**DOI:** 10.1155/2016/5024692

**Published:** 2016-10-30

**Authors:** María Eva Mingot-Castellano, Josefina Pérez-Núñez, Lourdes Baeza-Montañez

**Affiliations:** Hematology Department, Regional University Hospital of Malaga, Malaga, Spain

## Abstract

Factor V (FV) is a pivotal coagulation factor present in plasma and platelets. It plays an essential role in secondary haemostasis acting as a cofactor in the prothrombinase complex, catalysing the conversion of prothrombin to thrombin. There is little evidence on the management of mayor orthopaedic surgery in paediatric or adolescents subjects with this coagulopathy and almost no information about thromboprophylaxis in these situations. We report a case of a hip replacement in a 14-year-old girl with moderate FV deficiency (0.07 IU mL^−1^). As haemostatic replacement, inactivated fresh frozen plasma (FFP) was transfused at doses of 600 mL (15 mL kg^−1^, 45 kg weight) 2 hours before surgery and then sequential FFP infusions of 250 mL (7 mL kg^−1^) every 12 hours for 7 days. Plasma factor VIII, von Willebrand factor antigen, and von Willebrand ristocetin cofactor were monitored to avoid supranormal levels. Since the patient was sexually mature (Marshall and Tanner stage 5) with the hormone replacement therapy, she was immobilized and the surgery was considered as a high thrombotic risk. Thus, low molecular weight heparin was administered at doses of intermediate risk (Enoxaparin 20 mg daily, by weight) after finishing the daily infusion of plasma: 24 hours and during the 7 days after intervention. No tranexamic acid was used. No haemorrhagic or thrombotic adverse event was described.

## 1. Introduction

Factor V (FV) is a pivotal coagulation factor present in plasma and platelets. It plays an essential role in secondary haemostasis acting as a cofactor in the prothrombinase complex, catalysing the conversion of prothrombin to thrombin [1]. FV deficiency is an autosomal recessive bleeding disorder with a prevalence rate in the general population of 1 in 1 000 000 [2]. Bleeding symptoms vary from mild to severe, and mucocutaneous ones are the most frequent [1, 2].

There is little evidence on the management of surgery subjects with this coagulopathy [3]. Therefore, in order to add new information in this field, we report a case of a hip replacement in adolescence with moderate FV deficiency (0.07 IU mL^−1^).

## 2. Case Report

It is a case of a 14-year-old girl, with a history of left ureterocele and double pyelocaliceal system treated in the neonatal period with no bleeding complication during surgery. She suffered from a traumatic epiphysiolysis of her right hip at the age of 10 years in 2011. In March 2014, a right hip replacement was indicated. In preoperative coagulation studies, an abnormal prothrombin time (PT) and activated partial thromboplastin time (APTT) were detected. She was diagnosed with congenital FV deficiency, with FV plasma levels of 0.07 UI mL^−1^. As haemorrhagic manifestation, the patient presented menorrhagia with a Pictorial Blood Assessment Chart (PBAC) of 156 and symptomatic anaemia due to the iron deficiency. Since the patient presented a mild bleeding phenotype, a thrombophilia study was performed and the results obtained were normal (FV Leiden, mutation G20210A prothrombin gene, antithrombin, functional protein C, and total functional protein S). She started treatment with hormone replacement therapy and tranexamic acid during the first two days of menstruation as a measure of controlling bleeding and anaemia.

On November 2015, a right hip replacement for coxarthrosis secondary to epiphysiolysis was performed because of pain and difficulty to walk. The anaesthetic technique was general anaesthesia combined with analgesic ultrasound-guided femoral nerve blockade. As haemostatic replacement, inactivated (methylene blue) fresh frozen plasma (FFP) was transfused at doses of 600 mL (15 mL kg^−1^, 45 kg weight) 2 hours before surgery and then sequential FFP infusions of 250 mL (7 mL kg^−1^) every 12 hours for 7 days.  Table 1 shows the evolution of haemostatic parameters before and after surgery. Clinical evolution was good, with a bleeding profile comparable to the population without coagulopathy. About 550 mL of blood drains was recovered until the second postoperative day, with a complete cessation of bleeding on the third day after the intervention. No tranexamic acid was used.

**Figure 1:**
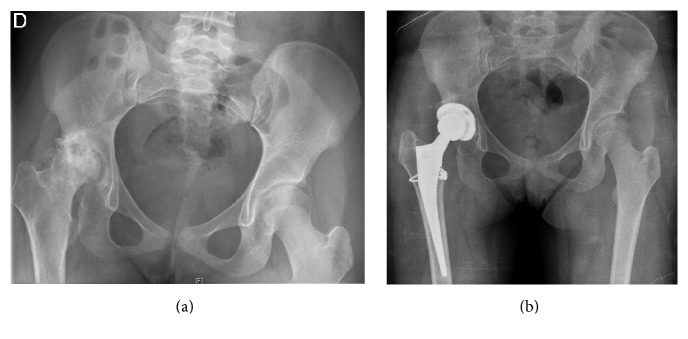
Simple radiology of hip before and after the intervention.

The patient was sexually mature (Marshall and Tanner stage 5) with hormone replacement therapy, she was immobilized, and the surgery was considered as a high thrombotic risk. Thus, low molecular weight heparin was administered at doses of intermediate risk (Enoxaparin 20 mg daily, by weight) from 24 hours after surgery to 7 days after intervention. Heparin was always injected after morning plasma infusion. Given the good clinical evolution and the absence of bleeding, the patient was discharged after 8 days after intervention. In Figure 1, the situation before and after surgery articulation is showed.

## 3. Discussion

Treatment of FV deficiency is limited by the absence of specific FV concentrates. Fresh frozen plasma (FFP) is the main treatment option, along with other possibilities such as platelet transfusions and activated recombinant factor VII (off-label) [2]. The therapeutic goal in case of invasive procedures and acute bleeding is FV levels above 0.2 IU mL^−1^. The procedure includes an initial infusion of inactivated FFC of 15–20 mL/kg, followed by 5 mL kg^−1^ every 12 hours, adjusting doses according to FV plasma levels, and the evolution of bleeding [6]. Platelet alpha-granules contain FV, that is the reason why platelet transfusions offer an additional haemostatic effect in this patient [2]. FV in transfused plasma may undergo rapid neutralization by an autoantibody or alloantibody. Factor V from platelet transfusion can help us to avoid this neutralization [7]. Recombinant-activated factor VII is licensed for the management of bleeding in patients with severe haemophilia and inhibitors and in treating bleeding in patients with severe platelet defects. There are reports of the off-label use of rFVIIa in FV deficient patients with and without inhibitors [3, 4]. The mode of action of rFVIIa makes it probable that haemostatic effects will be compromised in the absence of plasma/platelet FV. Activated prothrombin concentrate complex (aPCC) in association with platelets has been used anecdotally in patients with bleeds and FV inhibitor [7]. In Table 2, we describe all surgery reports in patients with FV deficiency found in literature. In our case, FV levels remained high over 0.2 IU mL^−1^ with good clinical response without increase of factor VIII (FVIII:c), von Willebrand antigen (VWF:Ag), and von Willebrand ristocetin cofactor (VWF:RCo) to thrombotic risk levels [8].

On the whole, extended pharmacological thromboprophylaxis is recommended in patients undergoing major orthopaedic surgery, from the day of surgery, in absence of bleeding [9]. There are no clear references regarding the thrombotic risk in adolescence under orthopaedic surgery and no one in subjects with FV deficiency. The prevalence of venous and arterial thrombosis is increasing in the paediatric and adolescent population [10, 11]. Although some authors describe as idiopathic up to 37% of cases of venous thrombosis in adolescent [12], others suggest the presence of two or more vascular risk factors in 81% of the adolescents with a thrombotic event [13]. Among these risk factors are obesity, immobilization, surgery, catheters, thrombophilia, hormone replacement therapy, smoking, and anatomical abnormalities. According to some authors, in adolescent subjects with vascular events, between 22% and 45% are immobilized, 18–27% are under surgery, and 5-6% are treated with hormone therapy [12, 13]. These risk factors were present in the patient we described, justifying the prescription of pharmacological thromboprophylaxis, despite the FV deficiency.

Since this case was a real challenge in terms of clinical patient management, due to the absence of mayor orthopaedic surgery in adolescents with FV deficiency in the literature, it would be crucial to determine algorithms to identify adolescents and coagulopathy patients with high thrombotic risk. Only then, it will be possible to adjust treatment in terms of antiplatelet or anticoagulant therapy in this population.

## Figures and Tables

**Table 1:** Haemoglobin and haemostasis parameters before surgery and after surgery.

	Before surgery	Day 0	Day +1	Day +2	Day +5	Day +7
Hb (gr dL^−1^)	11.6	11.4	9.6	10.1		9.2
Fibrinogen (grL^−1^)			3.6		5.5	
Factor V (IU mL^−1^)	0.07	0.42	0.32	0.44	0.44	0.31
Factor VIII (IU mL^−1^)	1.02	1.43	1.00	1.14	1.20	1.09
vWF:RCo (IU mL^−1^)	0.72	1.28	1.03	0.98	0.99	0.69
vW:Ag (IU mL^−1^)	0.85	1.32	1.00	0.87	0.90	0.85
D Dimer (ng mL^−1^)^*∗*^	150	1100	2400	1950	800	650

Hb: hemoglobin; vW:Ag: von Willebrand antigen; vW:Rco: von Willebrand ristocetin cofactor. _ _^*∗*^ELISA test.

**Table 2:** FV deficiency surgeries identified in literature.

Subject	Age	Basal Fv levels (IU mL^−1^)	Surgery	Treatment	FV postsurgery pick (IU mL^−1^)	Clinical outcome
1 [3]	6 days	Undetectable	Central line	(i) 15 mL kg^−1^ FFP TD 5 days, then OD 3 days, EOD till resolution.	0.32	Good
			Hickman	(ii) 19 mL kg^−1^ platelets, TD 2 days.		
				(iii) rFVIIa 90 mcg Kg^−1^ every 2 hours, 8 doses.		

1 [3]	5 months	Undetectable	Central line	(i) 15 mL kg^−1^ FFP TD 5 days, then OD 3 days, EOD till resolution.	No reference	Good
			Hickman	(ii) 19 mL kg^−1^ platelets, TD 2 days.		
				(iii) rFVIIa 90 mcg kg^−1^ every 2 hours, 8 doses.		

1 [3]	3 years	Undetectable	Tetralogy Fallot repairment	(i) 15 mL kg^−1^ FFP TD 2 days, then OD 11 days, (ii) 19 mL kg^−1^ platelets, TD 1 day, (iii) rFVIIa 90 mcg Kg^−1^, 1 dose, (iv) tranexamic acid^*∗*^.	0.39	Good

1 [3]	3 years	Undetectable	Central line	(i) FFP^*∗*^. (ii) Platelets^*∗*^.	No reference	Good
			Port-a-cath	(iii) rFVIIa 90 mcg mL^−1^, 2 doses.		
				(iv) Tranexamic acid^*∗*^.		

1 [3]	4 years	Undetectable	Central line	(i) FFP^*∗*^. (ii) Platelets^*∗*^.	No reference	Good
			Port-a-cath	(iii) Tranexamic acid^*∗*^.		
				(iv) rFVIIa 90 mcg mL^−1^, 2 doses.		

1 [3]	5 years	Undetectable	Central line	(i) FFP^*∗*^. (ii) Platelets^*∗*^.	No reference	Good
			Port-a-cath	(iii) rFVIIa 90 mcg mL^−1^, 2 doses. (iv) Tranexamic acid^*∗*^.		

2 [3]	6 weeks	Undetectable	Central line	(i) 15 mL Kg^−1^ FFP, TD 12 days, OD until resolution.	0.4	Good
			Hickman	(ii) 10 mL Kg^−1^ platelets, OD, 4 days.		
				(iii) rFVIIa 90 mcg Kg^−1^, 3 doses.		

2 [3]	8 months	Undetectable	Central line	(i) 15 mL Kg^−1^ FFP, TD 12 days, OD until resolution.	No reference	Good
			Port-a-cath	(ii) 10 mL Kg^−1^ platelets, OD, 4 days.		
				(iii) rFVIIa 90 mcg Kg^−1^, 3 doses.		

2 [3]	4 years	Undetectable	Central line	(i) 15 mL Kg^−1^ FFP, TD 12 days, OD until resolution.	No reference	Good
			Port-a-cath	(ii) 10 mL Kg^−1^ platelets, OD, 4 days.		
				(iii) rFVIIa 90 mcg Kg^−1^, 3 doses.		

2 [3]	6 years	Undetectable	Central line	(i) 15 mL Kg^−1^ FFP, TD 12 days, OD until resolution.	No reference	Good
			Port-a-cath	(ii) 10 mL Kg^−1^ platelets, OD, 4 days.		
				(iii) rFVIIa 90 mcg Kg^−1^, 3 doses.		

3 [3]	11 weeks	Severe	Craniotomy	(i) 15 mL Kg^−1^ FFP, TTD initial, progressive reducing. Total 10 days. (ii) rFVIIa 90 mcg Kg^−1^, 2 doses.	0.28	Good

3 [3]	12 weeks	Severe	Central line	(i) 15 mL Kg^−1^ FFP, TTD initial, progressive reducing.	No reference	Good
			Hickman	(ii) rFVIIa 90 mcg Kg^−1^, 2 doses.		

3 [3]	1 year	Severe	Central line	Not specified.	No reference	
			Port-a-cath			

4 [4]	60 years	0.15	Arthroscopic	(i) rFVIIa 120 mcg kg^−1^, 2 doses.	No reference	Good
			Synovectomy	(ii) 80 mcg kg^−1^ every 2 hours, 8 doses. (iii) Progressive reducing frequency 5 days. (iv) Tranexamic acid.		

4 [4]	60 years	0.15	Arteriography embolization	(i) rFVIIa 120 mcg kg^−1^, 2 doses. (ii) 80 mcg kg^−1^ every 2 hours, 6 doses. (iii) Progressive reducing frequency 3 days.	No reference	Good

5 [5]	27 years	0.05	Intrauterine insemination	FFP^*∗*^.	No reference	Good

^*∗*^No other information. EOD: every other day; FFP: fresh frozen plasma; OD: once a day; rFVIIa: recombinant factor VII activated; TD: twice a day; TTD: three times a day.
